# Unsupervised geochemical classification and automatic 3D mapping of the Bolshetroitskoe high-grade iron ore deposit (Belgorod Region, Russia)

**DOI:** 10.1038/s41598-020-74505-y

**Published:** 2020-10-20

**Authors:** Andrey O. Kalashnikov, Ivan I. Nikulin, Dmitry G. Stepenshchikov

**Affiliations:** 1 Geological Institute of Kola Science Centre of Russian Academy of Sciences (GI KSC RAS), 14 Fersman Street, Apatity, Murmansk Region, 184209 Russia; 2 Norilskgeologia Ltd, 11 Grazhdanskiy Pr., Saint Petersburg, 195220 Russia

**Keywords:** Economic geology, Geochemistry, Geology

## Abstract

We stated and solved three successive problems concerning automatization of geological mapping using the case of the Bolshetroitskoe high-grade iron ore deposit in weathered crust of Banded Iron Formation (Kursk Magnetic Anomaly, Belgorod Region, Russia). (1) Selecting a classification (clustering) method of geochemical data without reference sampling, i.e., solution of an “unsupervised clustering task”. We developed 5 rock classifications based on different principles, i.e., classification by visual description, by distribution of economic component (Fe_2_O_3_), by cluster analysis of raw data and centered log-ratio transformation of the raw data, and by artificial neural network (Kohonnen’s self-organized map). (2) Non-parametric comparison of quality of the classifications and revealing the best one. (3) Automatic 3D geological mapping in accordance with the best classification. The developed approach of automatic 3D geological mapping seems to be rather simple and plausible.

## Introduction

Theory and practice of spatial analysis in geosciences have been developing rapidly since the 1990s. However, geological maps and cross-sections are usually built manually. So, they significantly depend on a ‘human factor’. Recently, there is a trend to automatize a creation of geological maps and 3D models^1–5^. However, the ‘human factor’ takes place here too, e.g., it is required to create a priori rules, manually draw reference cross-section, etc. Plus, these methods are applied to relatively simple geological objects, i.e., gentle sedimentary strata or monotonous magmatic bodies.

Earlier, we proposed an approach of automatic 3D geological mapping based on interpolation and chemistry-to-mineral conversion and implicated it for an intricate magmatic (carbonatite-phoscorite) body^6^. A rock type in each cell of 3D space was ‘recognized’ on a basis of reference sampling, i.e., we had a set of samples with conjunct bulk-rock chemistry analyses and accurately determined mineral composition. So, it was a type of ‘supervised learning’ task. However, in geological practice, such reference sampling is often absent since it is impossible to exactly determine mineral composition of rocks (fine-grained or cryptocrystalline rocks, or strongly weathered rocks, etc.). In this work, we try to apply our approach to automatically map a geological object of this type without a reference sampling, i.e., it is an ‘unsupervised learning’ task. Furthermore, here we are studying a geological object of fundamentally another genesis, composition, structure, etc., namely, a high-grade iron ore deposit in weathered crust developed by banded iron formation (BIF).

Rocks inside a weathered crust formation are one of the most difficult rocks for mapping. These rocks are usually fine-grained (typical grain size is tens micrometers), in different physical state (from loose to rocky), significantly altered, complicated by relics and breccias, etc. So, these rocks are hard to classify by mineral (modal) composition, using both visual and microscopic investigation. Similar problems for volcanic rocks are solved in a general form, namely, classification by chemical composition by the TAS (total alkali–silica) diagram plus norm calculation by the CIPW^7,8^. There is no similar standard solution for sedimentary rocks in general and specifically for weathered crusts. So, we are forced to develop a classification ad hoc for such objects. On the analogy of the classification for volcanic rocks by chemical composition, we suggest that classification of the weathered crust rocks by chemical composition would be more relevant than by visual/optic microscopy investigation.

In statistics, this kind of problems is known as an “unsupervised clustering task”. For this task, a problem of quantitative comparison of different classifications quality is not solved in general form^9^, in contrast to a supervised learning task, where the clustering quality is estimated by cross-validation, bootstrap, etc.^10^. In this work, we suggest a particular method of quantitative comparison of classifications quality, however, we did not solve a problem of optimal quantity of clusters (i.e., rock types, in our case)—we used quantity of clusters defined by a visual geological description of drill core.

**Figure 1:**
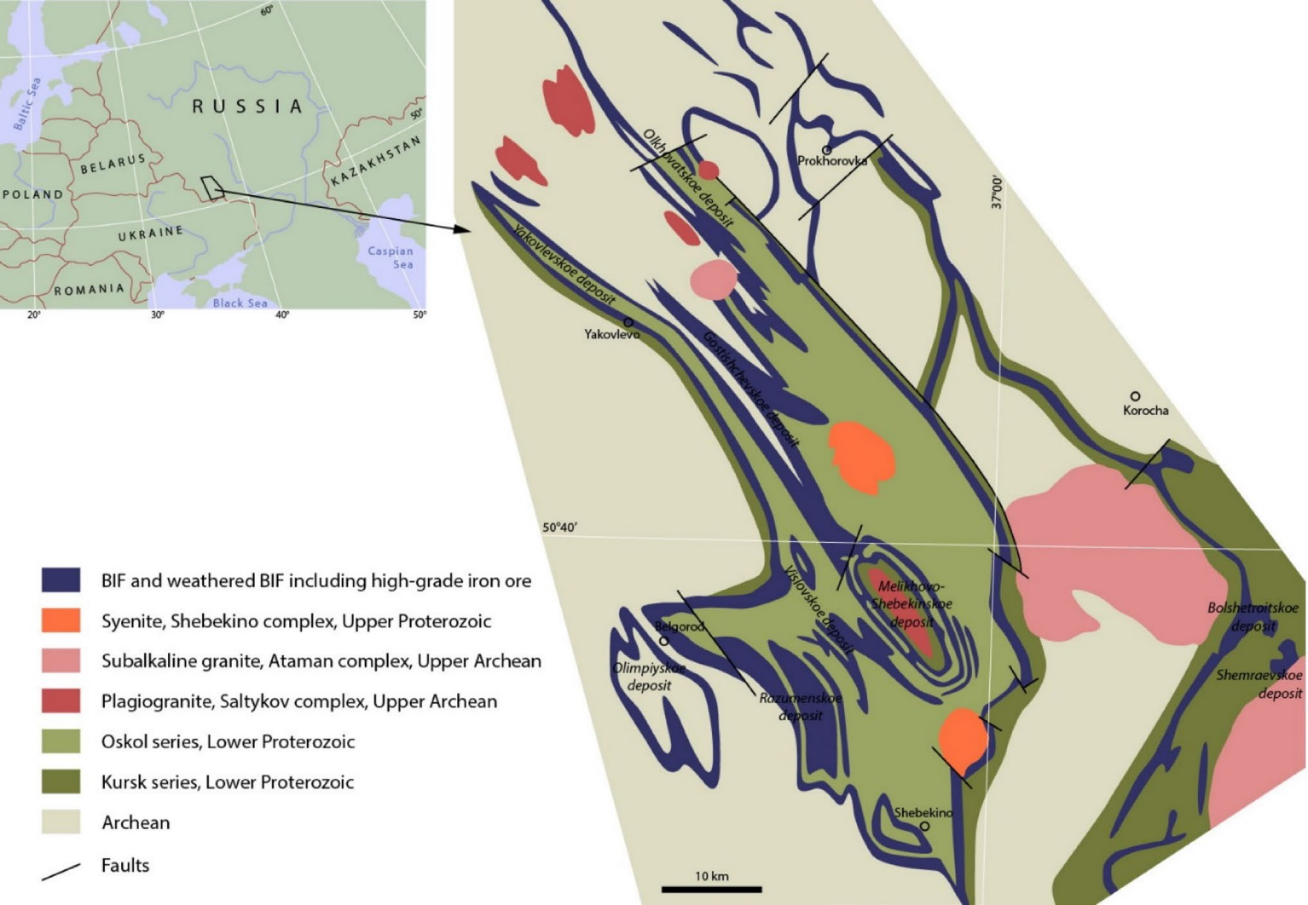
Geological map of the Belgorod iron ore district modified after^11–13^. Drawn by Adobe Illustrator CS6 (https://www.adobe.com).

**Figure 2:**
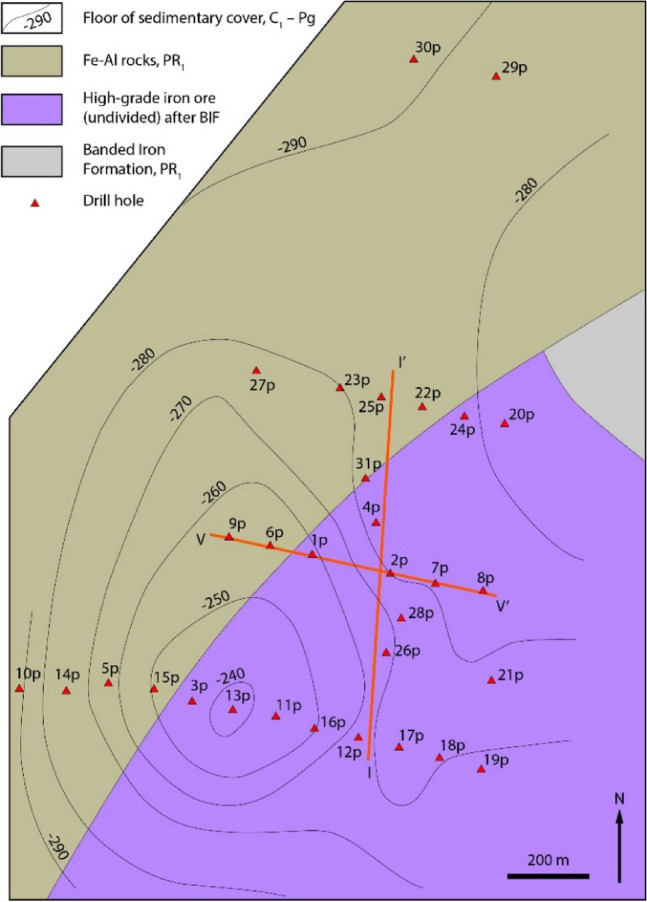
Geological map of the Bolshetroitskoe high-grade iron deposit, a surface under the sedimentary Phanerozoic cover modified after^17^. Orange lines are cross-sections in Figs. 5 and 6. Drawn by Adobe Illustrator CS6 (https://www.adobe.com).

We developed the method on the Bolshetroitskoe high-grade iron ore deposit in weathered crust of banded iron formation (BIF), Belgorod Region, Russia (Figs. 1, 2). In this paper, we developed several geochemical classifications of the deposit rocks based on different principles, i.e., visual description, one-dimensional statistics, multiple regression, and artificial neural networks. Then we introduced a method of quantitative comparison of classifications. Based on the best classification, we automatically built a 3D geological map of the deposit.

## Geological setting

The Bolshetroitskoe high-grade iron deposit is a part of the Belgorod ore district (Fig. 1). The Belgorod district is a world’s largest iron ore district. Beside the Bolshetroitskoe, there are the Yakovlevskoe, Gostishchevskoe, Shemraevskoe, Vislovskoe (measured resources), Razumenskoe, Olimpiyskoe, Melikhovo-Shebekinskoe, Olkhovatskoe (indicated and inferred resources) high-grade iron deposits in the district. All these deposits are large and unique.

High-grade iron ore of the Belgorod metallogenic district is considered to be a weathered crust of Banded Iron Formation (BIF) and, to a lesser degree, ferriferous schists of the pre-Visean age, the Carboniferous system, 346 ± 1 Ma^11^. The weathered crust rocks usually preserve structures and partly mineral composition of maternal rocks.

Main minerals of the high-grade ores are hematite (including martite—pseudomorph after magnetite, and microplaty hematite), goetite–limonite, magnetite, siderite, Fe-rich micas and hydromicas, quartz, clay minerals, boxites. When described visually, ore types are classified by proportions of these minerals, as well as by mechanical properties.

The Bolshetroitskoe high-grade iron deposit (Fig. 2) is located in the SW part of the Belgorod ore district and confined to a sharp bend of the Korochan–Mukhin regional magnetic anomaly. The Bolshetroitskoe deposit is considered to be a syncline, a part of the Belgorod regional graben-syncline. In a core of the Bolshetroitskoe syncline, there are Early Proterozoic BIF (Kursk series), and in limbs of the syncline, there are Archean rocks. High-grade iron ores of the Bolshetroitskoe deposit are considered to be formed in the Carboniferous weathered crust (pre-Visean age, 346 ± 1 Ma) after BIF of the Kursk series^14^.

The Bolshetroitskoe deposit was discovered in 1947 as a part of the Korochan–Mukhin regional magnetic anomaly. Research and development of a hydraulic borehole mining of the deposit was performed in 1988–1991. A detailed exploration was carried out in 2006–2013, and hydraulic borehole mining of the deposit took place by “Belgorodskaya GDK” (Belgorod, Russia) in 2008–2014. This innovative method allows mining loose rock from under a thick (~ 500 m) sedimentary cover^15,16^. Measured resources of the Bolshetroitskoe deposit are 410 Mt of ore and indicated resources are 2150 Mt of ore at Fe_total_ = 62.4%.

## Description of the approach

**Figure 3:**
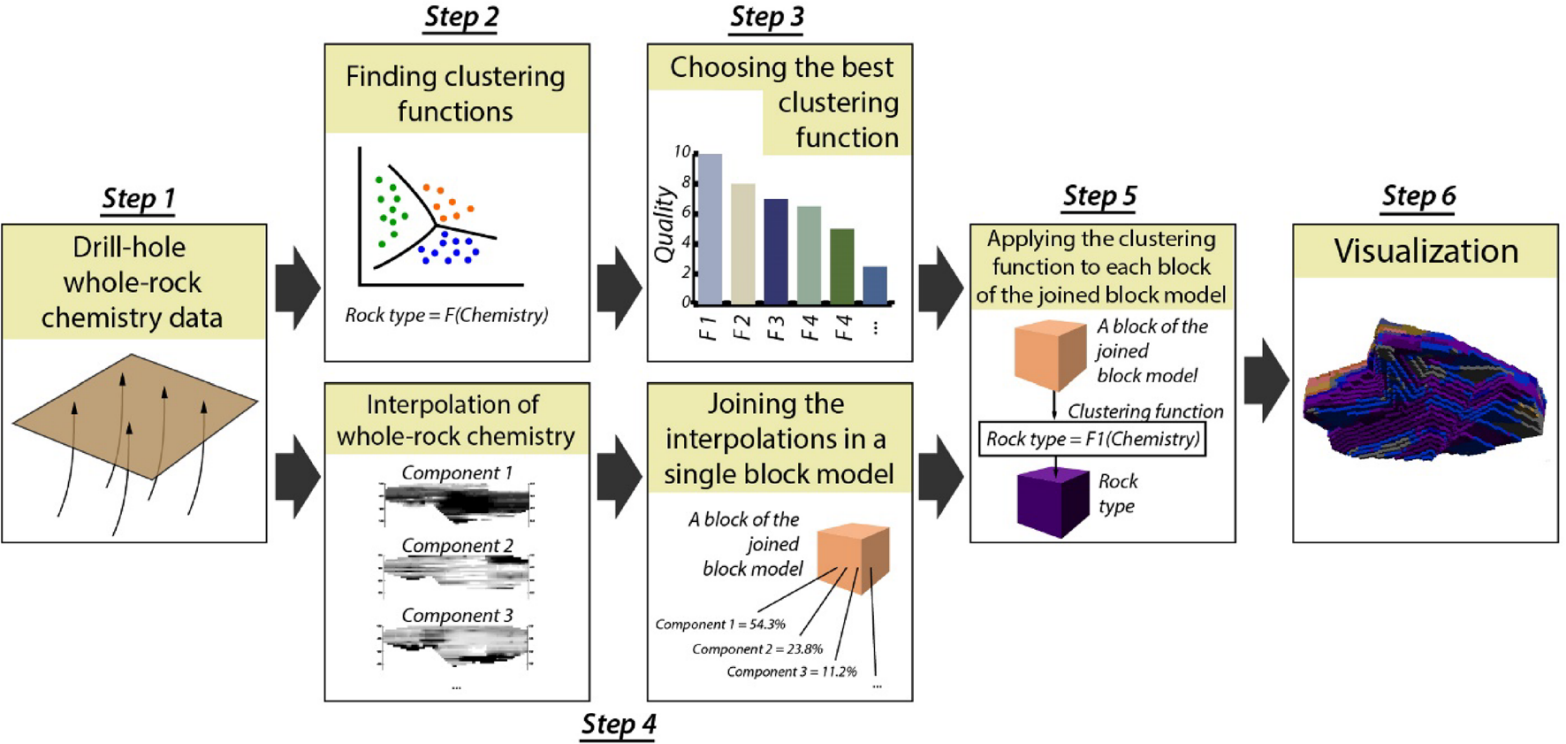
Flowchart of the automatic 3D geological mapping based on geochemical data without reference sampling.

The 3D automatic mapping of ore deposits without reference sampling consists in three general tasks: (1) Selecting a classification (clustering) method of geochemical data (“unsupervised clustering task”). (2) Interpolation of the input data. (3) Joining the results of the first two tasks, i.e., applying the selected clustering method to an interpolation block model. This block model will be a 3D geological map of a deposit. The more detailed approach is applied as follows (a flowchart is shown in Fig. 3).

- 1. Collecting and preparing representative data on whole-rock chemistry of a deposit in 3D.
- 2. Finding functions of data clustering by whole-rock chemistry (i.e., finding parameters of determination of rock type).
- 3. Choosing the best clustering function, if the best way of clustering is unknown beforehand.
- 4. Interpolation of whole-rock chemistry data taking part in the clustering. Joining the interpolation models in a single block model.
- 5. Applying the clustering function found in step 2 to each block of the single block model built in step 3, i.e., computation of rock type.
- 6. Visualization the computed rock type as a set of cross-sections, a 3D body, or a grid, etc.

Clustering and interpolation are different mathematical tasks, and comparison of classification is a task without a general solution, so we have placed solutions of the tasks for the Bolshetroitskoe deposit in separate subsections of the Results section. For usability, we have placed the number of the steps in the subsection titles.

## Results

### A general characteristic of the sample set (step 1)

**Figure 4:**
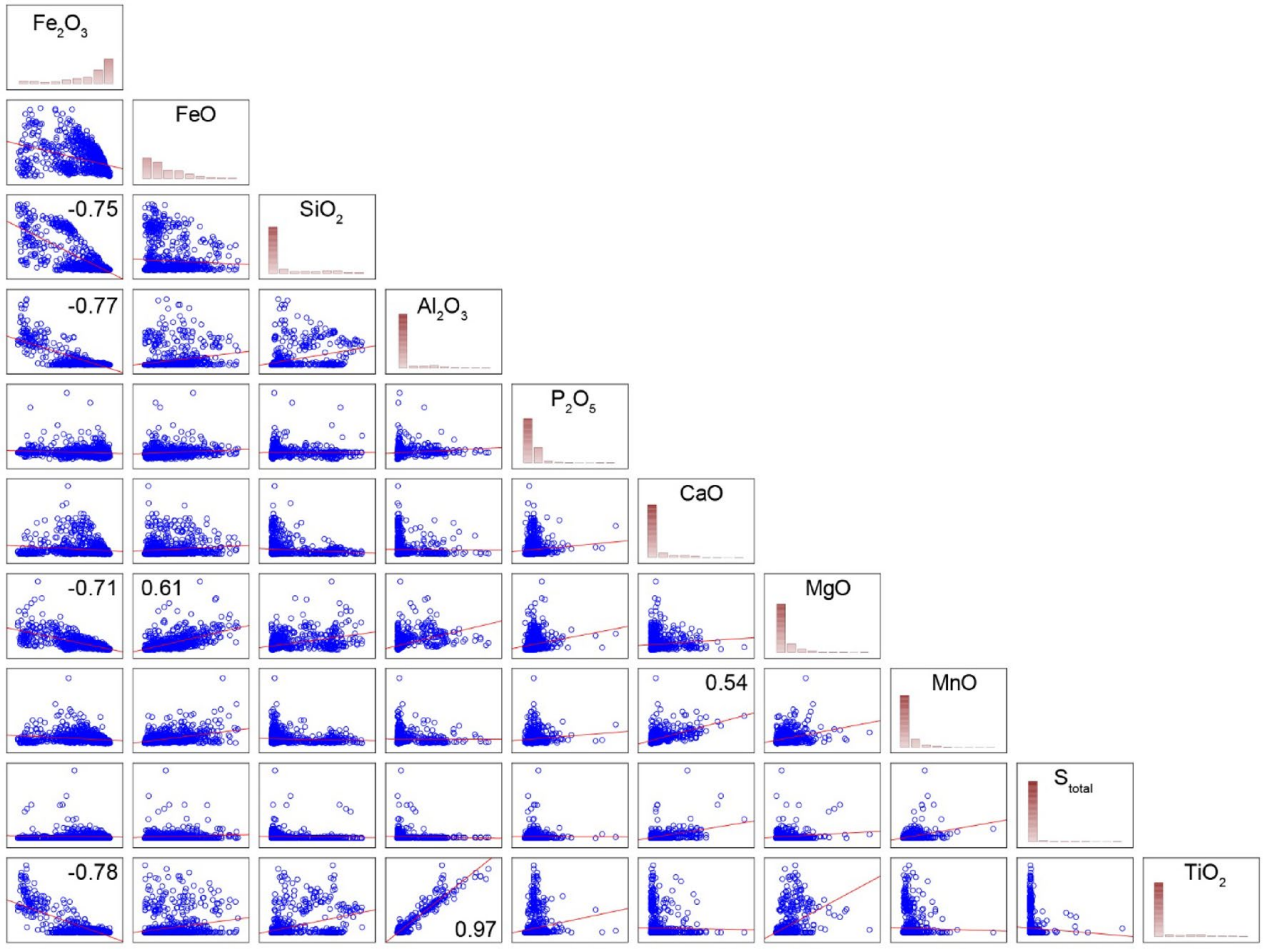
Scatterplots and histograms of the sampled population. Numbers are correlation coefficient *r* > 0.5 (*p* < 0.02).

1029 samples with an average length of 4 m were sampled along 28 drill holes (see details in the “Materials and methods” section). In the sample set, there are both ore of different quality and host rocks, so the set is obviously heterogeneous. Descriptive statistics of the sample set are given in Table 1, and correlations are shown in Fig. 4.

**Table 1:** Descriptive statistics of the sampled population.

	Mean	Geometric mean	Median	Minimum	Maximum	Lower quartile	Upper quartile	Percentile 5	Percentile 95	Standard deviation	Coef. variation
Fe_2_O_3_	72.23	64.16	83.10	1.50	96.64	61.20	89.22	13.26	93.93	23.93	33
FeO	7.42	5.56	5.45	0.55	29.58	3.29	10.60	1.47	18.98	5.60	76
SiO_2_	9.19	3.65	2.49	0.30	61.17	1.43	7.66	0.77	42.92	14.08	153
Al_2_O_3_	4.07	1.28	0.88	0.08	61.10	0.57	1.82	0.29	25.57	8.66	213
P_2_O_5_	0.103	0.093	0.100	0.016	0.840	0.073	0.110	0.042	0.190	0.059	57
L.o.i.	3.60	–	2.11	− 0.48	20.39	1.29	4.75	0.53	11.08	3.54	98
CaO	1.63	0.82	0.73	0.04	22.67	0.37	1.58	0.160	7.72	2.47	152
MgO	0.380	0.250	0.220	0.020	4.300	0.130	0.440	0.070	1.220	0.425	112
MnO	0.050	0.038	0.036	0.005	0.610	0.023	0.060	0.011	0.140	0.047	94
S_total_	0.173	0.079	0.081	0.010	7.200	0.034	0.160	0.011	0.570	0.401	231
TiO_2_	0.122	0.048	0.031	0.015	1.530	0.020	0.070	0.020	0.710	0.238	195

*L.o.i*. loss on ignition.

Strong verifiable correlation relationships are typical of Fe_2_O_3_ (with SiO_2_, Al_2_O_3_, MgO, TiO_2_). It is clear that these relationships are negative, i.e., the richer ore, the less impurities. Beside the component of interest, Fe_2_O_3_, there is a strong positive correlation of Al_2_O_3_ vs TiO_2_ (r = 0.97) and FeO vs MgO (r = 0.61). Nearly all scatterplots (even highly correlated Al_2_O_3_ vs TiO_2_) have minimum two trends, which is typical of heterogeneous samples.

Distribution of Fe_2_O_3_ is left-asymmetric (Q-normal), and the rest of the components have right-asymmetric distribution (lognormal or exponential).

### Geochemical classification of rocks (step 2)

To define the best approach to geochemical classification of the deposit rocks, we used four different methods, plus visual (‘manual’) geological classification as a basis for comparison.

**Figure 5:**
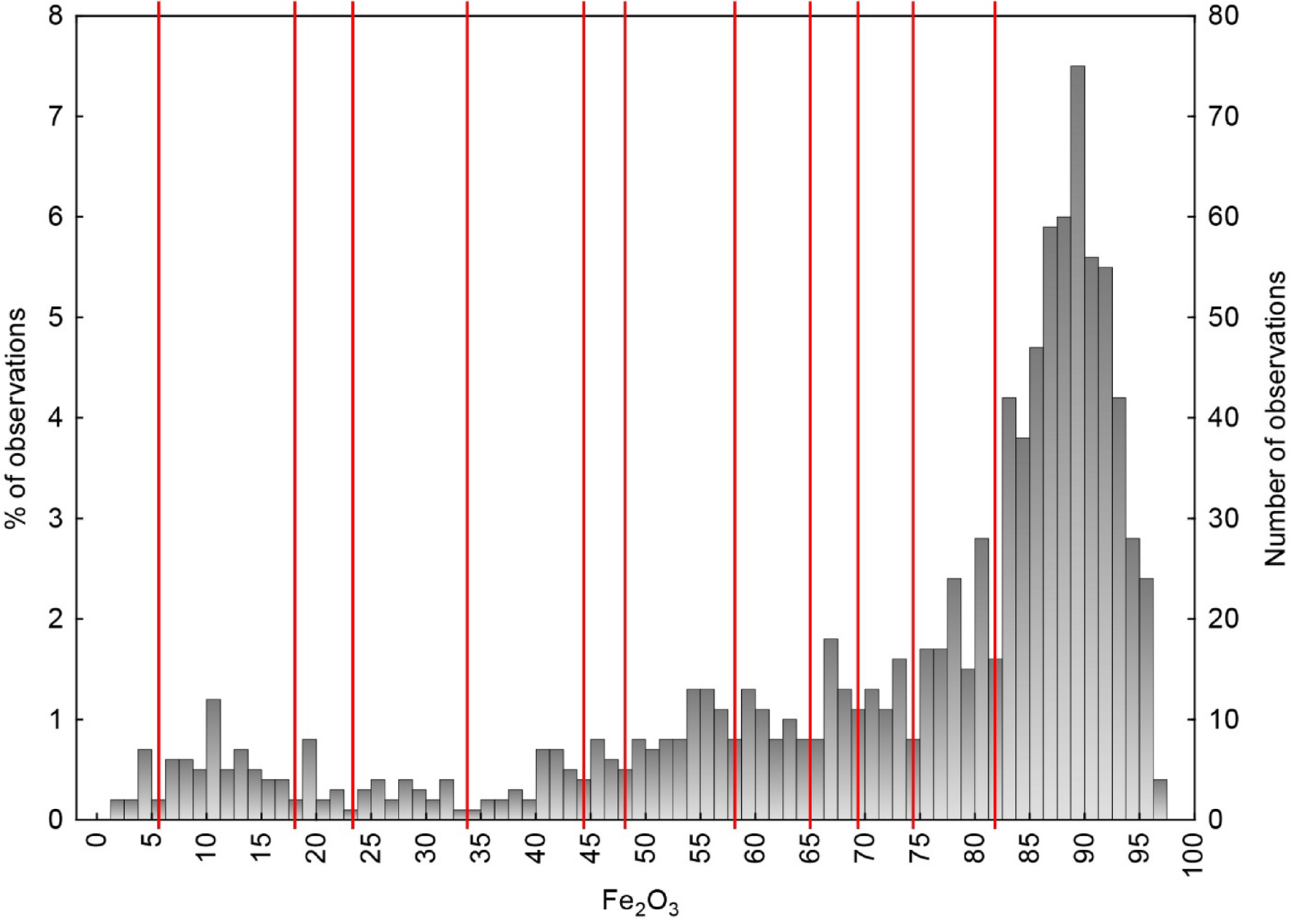
Histogram of Fe_2_O_3_ distribution in rocks of the Bolshetroitskoe deposit. Red lines are the borders of classes (local minima) of the Classification #2 (column 2 of the Table 2).

1. Geological classification of rock via visual description of a drill core by geologists of the “Belgorodskaya GDK” (Belgorod, Russia). They picked out 13 rock types: appreciably martite ore; banded martite ore; martite with magnetite and platy-hematite ore; appreciably platy-hematite limonitized ore; banded platy hematite limonitized ore with carbonate cement; martite with magnetite and platy-hematite ore with carbonate cement; appreciably limonite with hematite and martite ore; banded limonitized ore with silicates and carbonates; martite limonitized and sideritized ore with magnetite and platy-hematite; weathered intra-ore schist and allite; weathered above-ore schist; banded iron formation (BIF); breccia. In this work, we excluded the last rock type because it has no geochemical and mineralogical sense. So, we have 12 rock types, and in other classification, we picked out the same number of rock types.

2. Classification by content of the principal economic component, Fe_2_O_3_, based on its multimodal distribution (Fig. 5). We accepted local minima in the histogram as borders between rock types. This approach to ore classification is common since it suits for economic (technological) ore classification of single-component deposits (e.g., rich, intermediate, poor ores).

3. Cluster analysis of raw chemical composition. We used five rock-forming components: Fe_2_O_3_, FeO, SiO_2_, Al_2_O_3_, CaO. Method: k-means clustering. Initial cluster centers were taken by choosing observations to maximize initial between-cluster distances. Cluster: cases (rows). Number of clusters was 12, solution was obtained after three iterations. Missing data were casewise deleted.

4. Cluster analysis of chemical composition with centered log-ratio transformation of the raw data to avoid spurious correlation in closure numerical systems^18,19^. We used five rock-forming components: Fe_2_O_3_, FeO, SiO_2_, Al_2_O_3_, CaO. Method: k-means clustering. Initial cluster centers were taken by choosing observations to maximize initial between-cluster distances. k-means clustering. Initial cluster centers were taken by choosing observations to maximize initial between-cluster distances. Number of clusters was 12, solution was obtained after six iterations. Missing data were casewise deleted.

5. Clustering by artificial neural network (ANN). 5 rock-forming components were used: Fe_2_O_3_, FeO, SiO_2_, Al_2_O_3_, CaO. Type of ANN: Kohonnen’s network. Number of clusters was 12. Learning parameters: random sample sizes, train 70%, test 15%, validation 15%, seed for sampling is 1000, missing deleting handling (inputs) is casewise. Topological height 2, topological width 6. Comparison measure: Euclidian distance. 1000 training cycles. Learning rates: start 0.1, end 0.02. Neighborhoods: start 3, end 0. Normal randomization of network.

Table 2 shows results of the classifications: conventional name, and chemical composition of rock types. Rock types of different classifications (i.e., cells of a row of the table) did not correspond to each other.

**Table 2:** Description of geochemical rock types of the Bolshetroitskoe deposit picked out by 5 different classifications (classes in rows are independent).

	1. Manual	2. Histogram of Fe_2_O_3_	3. Cluster analysis of raw data	4. Cluster analysis of logratio transformed data	5. Neural network
1	Appreciably martite ore	Fe_2_O_3_* < 6%	Fe-Al rock	Medium to rich carbonatized ore	Rich ore
2	Banded martite ore	6–18%	Al-Si rock	Fe-Al-Si rock	FeO-bearing medium ore
3	Martite with magnetite and platy-hematite ore	18–23%	Al rock	Si-Fe-Al rock	Poor to medium carbonatized ore
4	Appreciably platy-hematite limonitized ore	23–34%	Fe-Si rock	Medium carbonatized ore	Poor ore
5	Banded platy-hematite limonitized ore with carbonate cement	34–44%	Carbonatized Fe-Al rock	Aluminous Fe ore	Banded iron Formation
6	Martite with magnetite and platy-hematite ore with carbonate cement	44–48%	Rich ore	Medium to rich ore	Si-Fe-Al rock
7	Appreciably limonite with hematite and martite ore	48–58%	Poor carbonatized ore	Carbonatized Fe rock/poor ore	Moderately weathered BIF
8	Banded limonitized ore with silicates and carbonates	58–65%	Si rock/quartzite	Rich ore	Medium to rich ore
9	Martite limonitized and sideritized ore with magnetite and platy-hematite	65–69%	Medium ore	Al-Si rock	Poor to medium siliceous aluminous ore
10	Weathered intra-ore schist and allite	69–74%	Poor siliceous ore	Carbonatized Si rock/Poor siliceous ore	Al–Fe rock
11	Weathered above-ore schist	74–82%	Fe-Al-Si rock	Carbonatized ore	Si-Al–Fe rock
12	Banded iron formation	> 82%	Carbonatized poor ore	Poor siliceous ore	Al-Si rock

### Comparison of the classifications (step 3)

As we obtained five classifications of the same object, and these classifications are based on different principles, a problem to choose the best classification raised.

Method of comparison of approximation and interpolation is known in statistics, e.g., cross-validation^20^ and bootstrap^10^. However, approximation and interpolation problem differs from clustering (classification) problem. It has a reference sample set, and measure of fitting quality is based on comparison of the reference sample set and approximation/interpolation model. Sometimes there is a reference set for classification problems, it is known as a supervised learning task, see review in^21^. However, we did not have a reference set, i.e., we had an unsupervised learning task. For such type of problems, there is no general solution, and the method of quantitative comparison of classifications is usually developed for a specific problem, see review in^9^. Statement of the problem in a general form and an approach to its solution were introduced in^22^. Our case was simpler because we did not take into account the problem of selection of cluster number. We took 12 rock types since this number of rock types (excluding the ‘breccia’ type) is used for the Bolshetroitskoe deposit, and the approximately same number of rock types is used for other high-grade iron ore deposits of the region.

In accordance with the approach suggested by Dy and Brodley^22^, we supposed that a sum of ‘inhomogeneity’ of all classes (from 1 to 12) of the classification *m* could be a measure of negative quality *Q*_*m*_. I.e., the less sum of ‘inhomogeneity’ of all classes, the better a classification under the condition that a number of classes is equal in all compared classifications. Thus, the optimal classification has a minimal sum of ‘inhomogeneity’ Q^*^:

1 $$Q^{*} = \mathop {\min }\limits_{{m = \overline{1,5} }} Q_{m}$$

This approach is in agreement with a definition of clustering as grouping of similar objects^23^. We used the standard deviation σ as a measure of inhomogeneity.

A flowsheet for comparing the quality of classifications (in our case) is as follows.

1. Calculate standard deviation $\sigma_{ij}^{m}$ of each component *j* ($j = \overline{1,\,5}$) in each class *i* ($i = \overline{1,\,12}$) of each classification *m* ($m = \overline{1,\,5}$).
2. Calculate sum $Q_{j}^{m}$ of the standard deviations of all components for each class of each classification:
  2 $$Q_{j}^{m} = \sum\limits_{i = 1}^{12} {\sigma_{ij}^{m} }$$
3. Based on the sum $Q_{j}^{m}$ of the standard deviations, determine rate $r_{j}^{m}$ of each classification for each component: the less sum, the higher rate (in our case, $r_{j}^{m}$ = 1 for minimum $Q_{j}^{m}$, and $r_{j}^{m}$ = 5 for maximum).
4. Calculate sum of rates $S^{m}$ of all components for each classification:
  3 $$S^{m} = \sum\limits_{j = 1}^{5} {r_{j}^{m} }$$
5. Determine final rates *R*_*m*_ of the classifications: the less sum, the higher rate (in our case, *R*_*m*_ = 1 for minimum *S*^*m*^, and *R*_*m*_ = 5 for maximum). These final rates reflect comparative quality of the classifications.

A result of application of the flowsheet to the sample set of the Bolshetroitskoe deposit is shown in Table 3.

**Table 3:** A comparison of quality of five geochemical classifications (see Table 2) of rocks of the Bolshetroitskoe deposit. σ is a standard deviation of the component of the rock type.

Rock type	1	2	3	4	5	6	7	8	9	10	11	12	Sum	Rate	Rate sum/final rate
**1. Manual classification**															
N	173	105	89	77	42	37	71	90	71	44	63	110	972		20/4
σFe_2_O_3_	5.93	8.71	10.68	8.38	8.28	15.87	7.83	7.30	13.96	20.10	15.16	15.73	137.93	5	
σFeO	3.62	3.92	6.08	4.10	3.56	6.32	2.79	3.45	6.66	7.65	7.62	3.99	59.76	4	
σSiO_2_	1.79	4.03	3.77	2.42	4.52	4.72	4.99	3.28	2.54	15.22	14.96	13.44	75.68	4	
σAl_2_O_3_	0.79	1.78	1.24	1.24	0.61	3.66	0.67	0.61	0.81	9.74	12.17	9.24	42.57	3	
σCaO	1.02	1.47	3.43	2.43	2.66	1.81	1.99	1.19	3.33	2.56	2.32	3.39	27.60	4	
**2. Classification by content of economic component (Fe**_**2**_**O**_**3**_** )**															
N	12	56	15	23	32	17	75	54	40	50	120	535	1029		21/5
σFe_2_O_3_	1.26	3.13	1.31	2.74	2.61	1.04	2.74	1.99	1.05	1.40	2.19	3.52	24.98	1	
σFeO	4.15	8.28	9.18	6.49	6.80	8.25	7.28	6.46	5.26	5.18	3.90	2.68	73.90	5	
σSiO_2_	20.26	15.50	13.98	14.99	16.54	19.11	17.24	12.18	7.38	6.22	4.10	1.74	149.24	5	
σAl_2_O_3_	13.52	11.06	8.43	10.17	5.14	5.42	6.18	5.40	1.45	1.95	0.92	0.39	70.02	5	
σCaO	0.29	1.57	2.88	2.65	2.59	4.42	4.16	3.43	3.82	3.79	2.43	0.94	32.96	5	
**3. Classification by cluster analysis of raw data**															
N	12	25	11	39	26	418	120	25	230	56	29	38	1029		12/2.5
σFe_2_O_3_	5.22	5.34	2.66	6.30	5.97	2.75	4.08	5.28	3.32	4.93	6.83	6.13	58.80	2	
σFeO	3.89	4.75	4.19	3.87	4.29	1.89	4.86	2.51	3.55	1.28	5.25	5.49	45.83	2	
σSiO_2_	5.38	5.07	5.57	3.40	6.36	1.19	4.98	5.08	3.59	5.46	5.53	2.65	54.28	1	
σAl_2_O_3_	5.52	4.06	8.07	2.46	8.05	0.36	2.17	4.41	0.69	0.63	4.35	2.59	43.36	4	
σCaO	3.77	0.35	2.74	0.60	3.79	0.84	3.74	0.56	2.11	1.64	1.79	4.99	26.91	3	
**4. Classification by cluster analysis of logratio transformed data**															
N	171	33	44	84	75	186	42	167	42	60	74	51	1029		12/2.5
σFe_2_O_3_	4.79	16.61	8.59	10.80	12.04	5.20	9.56	3.47	9.66	15.20	12.24	11.00	119.16	4	
σFeO	1.63	6.45	6.47	5.13	5.57	3.56	4.03	1.80	2.18	3.27	6.65	1.29	48.03	3	
σSiO_2_	1.40	6.24	9.43	1.72	2.38	0.56	8.70	1.13	9.54	11.52	0.74	10.03	63.40	3	
σAl_2_O_3_	0.40	10.96	12.10	1.61	4.53	0.33	0.28	0.34	6.81	0.94	0.50	0.79	39.58	1	
σCaO	1.44	2.72	0.32	1.90	0.31	0.21	3.10	0.23	0.48	1.39	3.94	0.23	16.28	1	
**5. Classification by neural network**															
N	241	143	48	39	89	25	42	220	93	18	31	40	1029		10/1
σFe_2_O_3_	2.72	4.08	8.28	8.02	8.95	6.93	6.18	2.71	6.91	11.62	7.94	5.75	80.09	3	
σFeO	0.93	1.46	4.34	3.43	3.66	3.02	2.15	1.09	2.03	3.51	4.37	2.39	32.37	1	
σSiO_2_	1.42	1.69	4.66	2.65	4.66	10.74	5.45	1.42	3.76	7.17	6.75	8.08	58.44	2	
σAl_2_O_3_	0.32	1.25	1.13	2.48	1.75	11.58	1.25	0.49	3.10	7.09	5.62	6.25	42.31	2	
σCaO	0.94	0.81	2.87	3.08	0.91	1.81	1.53	1.13	1.43	3.57	1.88	0.51	20.46	2	

N is a number of samples in the class. σ is a standard deviation of the responding component, as in Eq. (2).

The result shows that the classification by neural network is the best. The neural network is available online in Supplementary Materials 1. So, this classification became a basis for a 3D automatic geological mapping of the Bolshetroitskoe deposit. A chemical composition of the rock types picked out by this classification is shown in Table 4.

**Table 4:** Mean chemical composition of the rock types of the Bolshetroitskoe deposit picked out by artificial neural network (Classification #5).

	Rock types in accordance with Table 2 (classification #5. Neural network)											
	1	2	3	4	5	6	7	8	9	10	11	12
N	241	143	48	39	89	25	42	220	93	18	31	40
Fe_2_O_3_	91.65	81.97	65.72	54.80	48.55	12.22	75.07	87.23	68.90	43.50	20.40	10.88
FeO	2.50	10.02	7.39	21.21	5.47	11.00	3.89	5.45	14.50	8.08	20.99	3.62
SiO_2_	1.95	2.39	3.80	3.25	40.33	19.72	15.36	2.37	4.26	8.24	20.23	47.96
Al_2_O_3_	0.69	1.31	1.16	2.87	1.10	39.06	1.25	0.96	3.42	23.02	21.79	22.98
P_2_O_5_	0.09	0.10	0.10	0.13	0.09	0.11	0.11	0.10	0.11	0.12	0.14	0.11
L.o.i.	1.72	1.96	9.23	10.31	1.99	12.31	2.24	1.87	4.82	9.84	9.60	6.25
CaO	0.96	0.75	10.23	3.51	1.08	1.06	1.30	1.09	1.48	4.25	1.69	0.65
MgO	0.13	0.27	0.35	0.94	0.55	0.79	0.23	0.20	0.55	0.57	1.44	0.97
MnO	0.03	0.05	0.12	0.14	0.04	0.04	0.03	0.04	0.08	0.09	0.06	0.04
S_total_	0.10	0.17	0.86	0.42	0.05	0.06	0.13	0.13	0.19	0.09	0.10	0.27
TiO_2_	0.03	0.05	0.05	0.07	0.04	0.99	0.06	0.04	0.09	0.58	0.68	0.69

### Interpolation, rock type evaluation, and visualization of the 3D geological model (steps 4–6)

Application of the automatic 3D geological modelling method to the Bolshetroitskoe deposit (steps 4–6 in accordance with the flowchart, Fig. 3) is described below.

Step 4.1. Interpolation of the determinative components: Fe_2_O_3_, FeO, Al_2_O_3_, SiO_2,_ and CaO.

**Figure 6:**
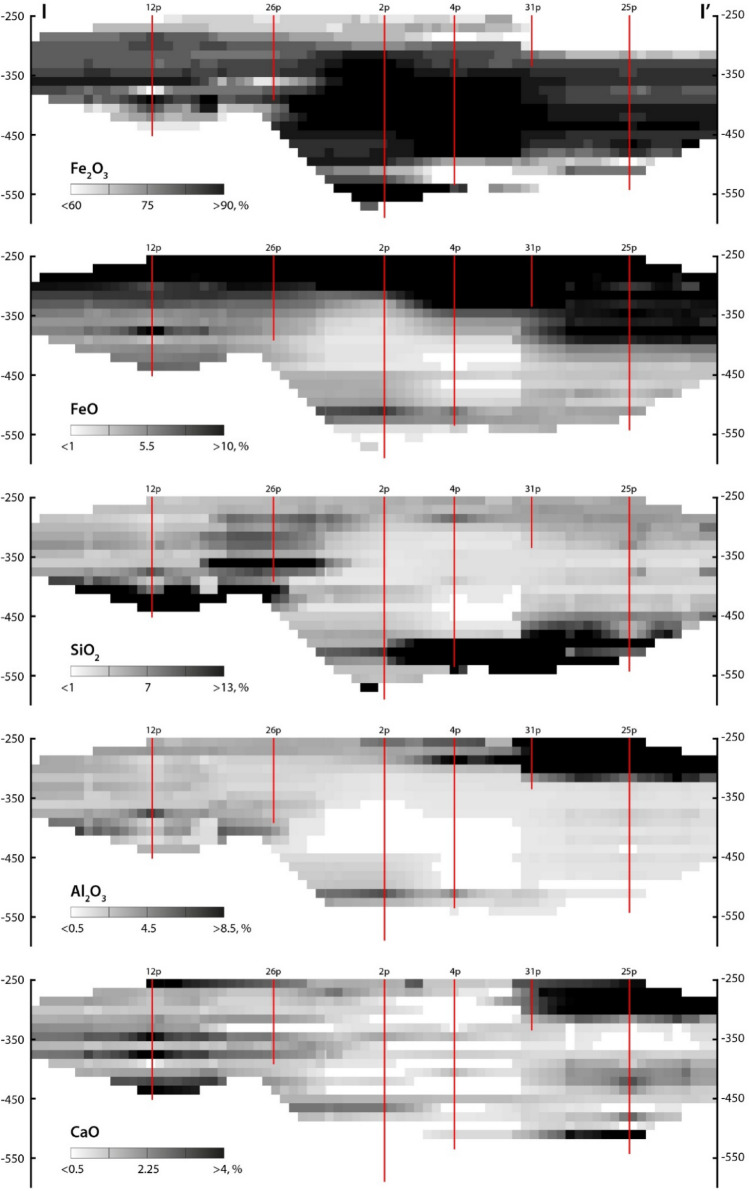
Distribution of rock-forming components in the cross-section I–I’ in Fig. 2. Red lines are drill holes.

The interpolation was conducted using the anisotropic inverse distance weighted method, power = 2. Search ellipsoid was determined by variography (set of directional semivariograms) of the dominant component Fe_2_O_3_, taking into account distances between boreholes. Interpolation of each component was carried out in three runs with a successive increase in the search radius (130, 270, and 560 m) and a decrease in the threshold number of points falling into the search ellipsoid (4, 3, and 1). A number of sectors of the search ellipsoid is 4, a maximum number of points in a sector is 5. Parameters of the search ellipsoid: azimuth of the 1st axis is 90°, dip is 0°, factor is 1; azimuth of the 2nd axis is 180°, dip is 0°, factor is 1; azimuth of the 3rd axis is 0°, dip is 90°, factor is 0.1. Sections of the interpolation block models of the five components across the line I–I’ (Fig. 2) are shown in Fig. 6.

Step 4.2. Conjugation of the interpolation block models into one table. As a result, we have a block model with value of each chemical component for each block.

Step 5. Evaluation of a rock type for each block using the previously created classification [see the sections ”Geochemical classification of rocks (step 2)” and ”Comparison of the classifications (step 3)”], i.e., clustering by artificial neural network (Kohonnen’s self-organized map), the program code of which is presented in the Supplementary Material file.

**Figure 7:**
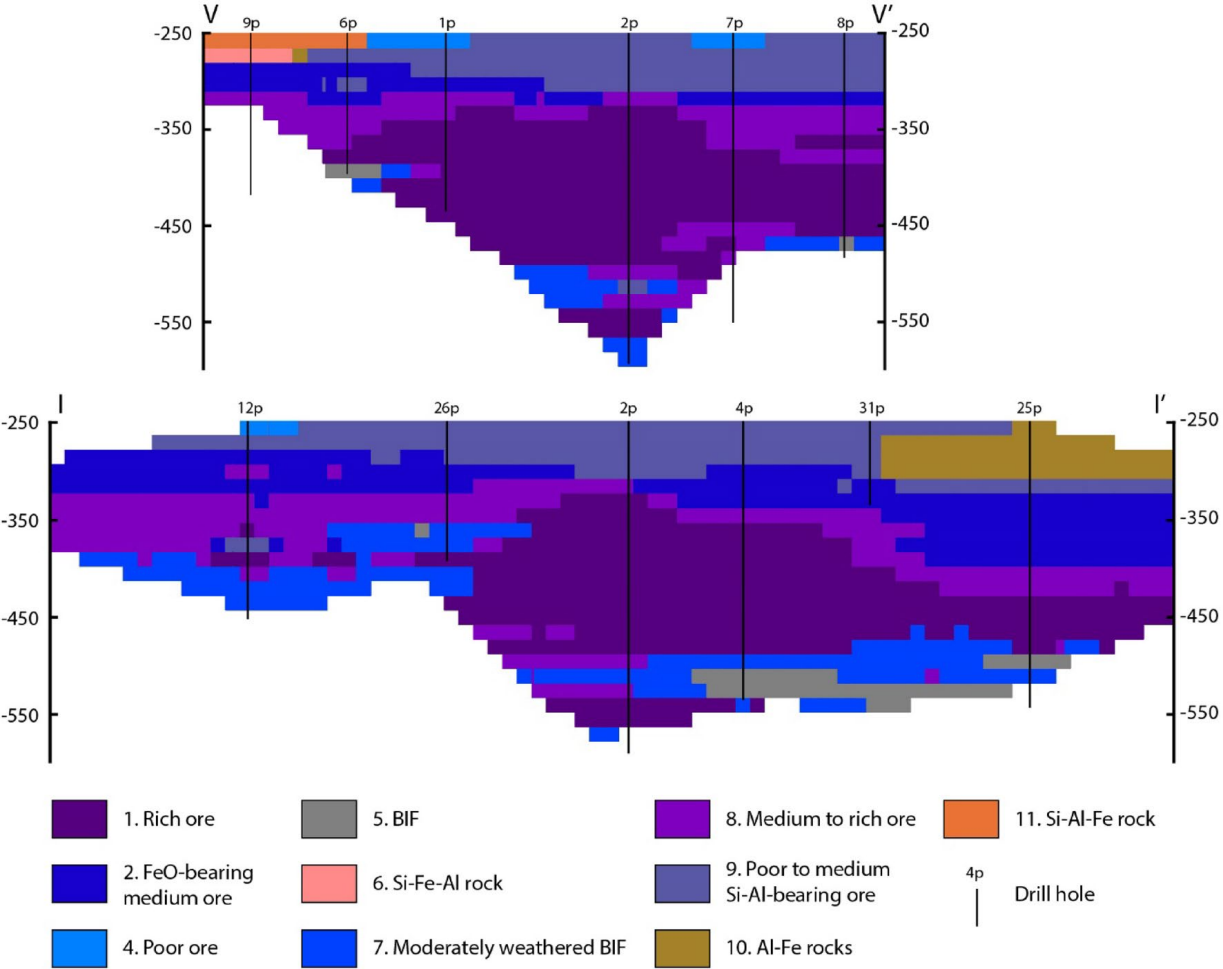
Cross-section of the automatically built 3D geological model of the Bolshetroitskoe deposit across lines V–V’ and I–I’ showed in Fig. 2. Numbers of rock types in the legend correspond to Tables 2, 3 and 4.

Step 6. Visualization of the block model. The final result is shown in Fig. 7.

## Discussion

### Model of the Bolshetroitskoe deposit

**Figure 8:**
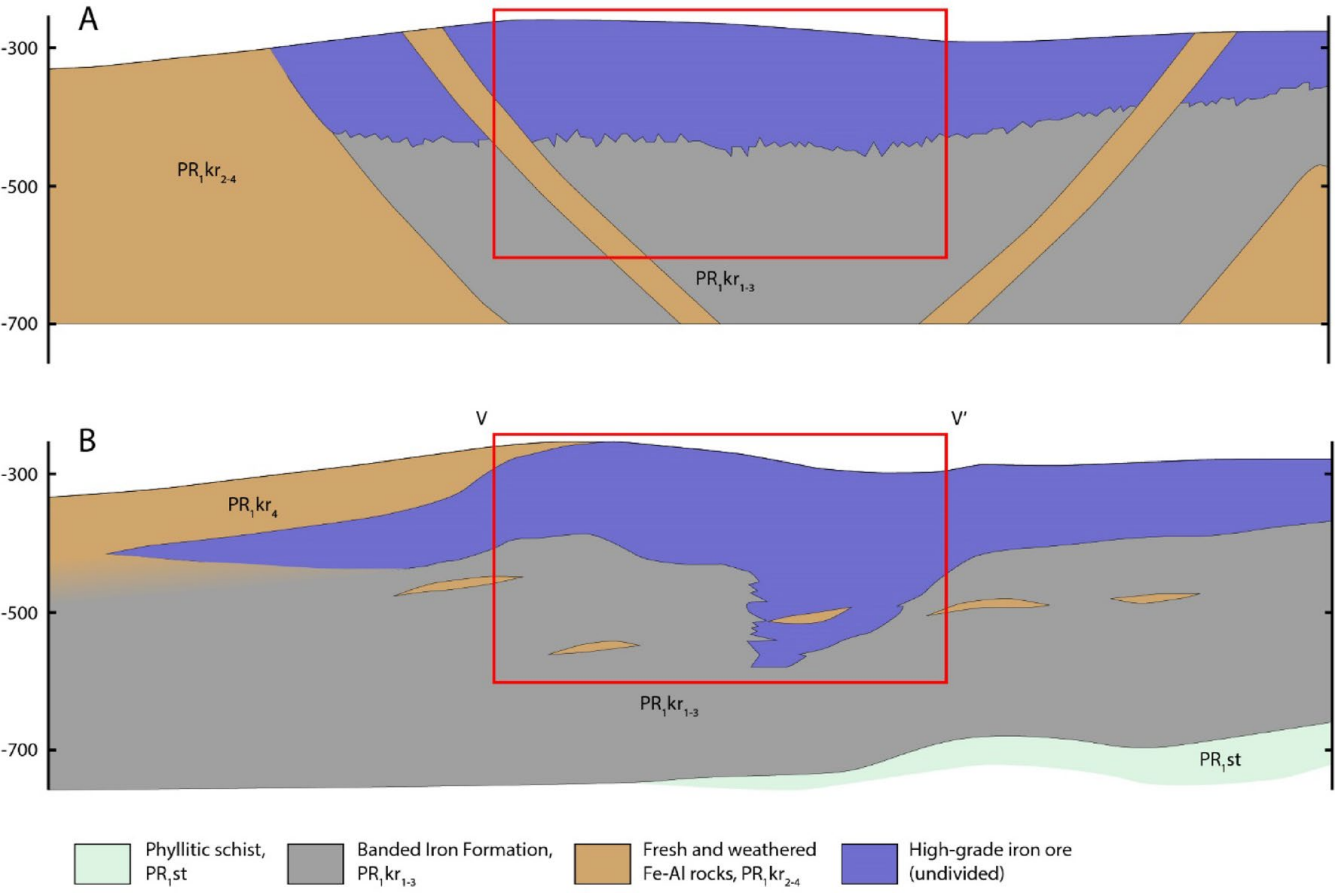
Manually built cross-sections of the Bolshetroitskoe deposit (without detailed ore and rock types). **(A)** Built by V.N. Klekl (modified after^24^); **(B)** built by M.M. Bezugly and I.I. Nikulin (modified after^15^). Red rectangle corresponds to the cross-section V–V’ in Fig. 7.

We got the 3D geological model of the deposit without human decisions. It is based only on structure of spatial variation of rock-forming components (Fe_2_O_3_, FeO, Al_2_O_3_, SiO_2_, CaO). The spatial variation cannot be utilized during manual drawing of geological model or cross-section of a deposit. Plus, geologists usually have a priori model of genesis and a structure of a deposit that can influence on a geological model. For example, there are two manually built cross-sections of the Bolshetroitskoe deposit (Fig. 8)^15,24^. We can see that the authors of the first cross-section had a conception that the regional folding formed the deposit, and the authors of the second one supposed subhorizontal bedding with tectonically induced permeability, which resulted in a thick zone of the high-grade ore. An automatic mapping approach is data-driven and free of any a priori conception. These two circumstances (basing on spatial variation and absence of a priori conception) forced us to suggest that the automatic approach can be more precise than the manual one. A general agreement of our automatic model and the most recent manual cross-section (Fig. 8b) supports this suggestion.

However, the automatically built geological model has some constrains or shortcomings.

– Some rock types are named as formation, viz. BIF, and moderately weathered BIF. Of course, they are conditional names since they should be referred to a certain formation based on textural, structural, and mineral properties. Yet, in our case, we use these names for more lucidity because it usually coincides with a visual description of drill cores.

– Most of rock types are named in accordance with their chemical composition, and these names does not have geological sense of the rock types. We suppose that it is the main shortcoming of the developed geological model. However, the shortcoming arises from quality of input data, in this case. If we obtained an exactly determined mineral composition and quantitative description of textural/structural properties throughout the drill cores, we would build a geologically interpretable model, as we did in the works^25,26^.

– Classification of some blocks seems to be wrong from the geological point of view. E.g., near the bottom of the drill hole 26p (Fig. 6), there is a block of BIF surrounded by a moderately weathered BIF above high-grade ore blocks.

In general, the automatically built structure of the deposit seems to be geologically correct: BIF and moderately weathered BIF are in the bottom of the cross-section; there are iron ores of different grades above maternal BIF; and, finally, iron ores are overburden by silica- and alumina-rich rocks; the highest-grade ore (rock type #1) forms the central bulge (around drill hole 2p).

### Comparison of the classifications of rocks of the Bolshetroitskoe deposit

It was foreseeable that the one-parameter classification by Fe_2_O_3_ histogram became the worst one, its rate sum is twice worse than the leader’s, the classification by ANN (21 vs 10, Table 3). Surprisingly, the manual classification became the worst too (rate sum is 20), and logratio transformation of raw data did not enhance the classification: rate sum of the classification by cluster analysis of raw data is 12, and logratio transformed is 12, too (Table 3). Clustering of the data by artificial neural network became the best (rate sum is 10). It is expectable because it is known that multiparametric non-linear methods of clustering are better than k-means cluster analysis, see e.g.^27,28^.

We used standard deviation as *σ* in Eq. (2) since it is the most common measure that is used to quantify the degree of variation of a data value set, although other measures such as interquartile range, median absolute deviation, mean absolute difference, average absolute deviation, etc. can be used. In our case, we tested other ‘measures of unhomogenity’ (interquartile range and weighted standard deviation) and found that the classification by artificial neural network is still a leader. Justification of the best ‘measure of unhomogenity’ and its usability condition is an independent mathematical problem and is outside the scope of this work. Rigorous mathematical investigation and generalization of the approach are in our future plans.

### Approach of automatic 3D geological mapping

In general, the approach consists of the following three steps: (1) interpolation of variables required for rock type determination in a single block model; (2) rock type determination for each block of the block model by a certain classification algorithm; (3) visualization. The most difficult problem is step 2. A classification algorithm depends on available data. The ideal (or simplest) case is when a directly determined mineral composition and quantified textural properties of rocks are available^25,26^, the classification algorithm will be just a logical evaluation of rock type in accordance with the commonly accepted classification (as in our aforementioned works) or any local classification. The case is more complex when a mineral composition is calculated from a chemical composition of rocks^6^, i.e., a chemistry-to-mineral conversion problem^29,30^ should be taken into account. The most complex case—rock classification by “plain” rock chemistry—is investigated here. In all these cases, the principal workflow is the same. It suggests that the developed approach is sufficiently universal. It seems that the most difficult problem (rock classification algorithm) can be reduced by total determination of mineral composition during a deposit exploration, e.g., by automatized mineralogical systems like QEMSCAN^29,31^. The second important source of errors of the geostatistical-based approach is interpolation. However, this field of knowledge is being actively developed now, and nearly all problems of uncertainty usually have an acceptable solution, general or special (see review e.g., in^32,33^).

Except for a chemistry-to-mineral conversion problem, the developed approach does not require a specially developed mathematical software or code. Commonly used mining or geographical information systems (e.g., Micromine, Datamine, Mineframe, or ArcGIS, etc.) are suitable for implementing the approach.

One more feature of the approach is a requirement of quantified data (mineral and chemical composition of rock, quantified structural/textural properties, etc.), and qualitative data (e.g., visually described textural properties) cannot be used since such type of data cannot be interpolated. We suppose that it is an advantage because an interpolation-based analysis of spatial structure of a deposit is more founded than intuitively drawn manual cross-section (see the example in Fig. 8). Besides, the approach can be used for a geometallurgical modelling: in this case, technological ore types should be taken into account (determined by mineral processing or metallurgical technology) instead of common geological rock typessup^34^.

## Conclusions

- 1. We developed an approach of rock type classification and 3D automatic mapping of ore deposits without reference sampling (i.e., by unsupervised learning). Methods of non-linear clustering are preferable for rock type classification without reference sampling (e.g., Kohonnen’s self-organizing map).
- 2. We introduced a method of non-parametric comparison of quality of classifications based on different principles. The method is to rank classifications by a sum of standard deviations within classes.
- 3. Interpolation of rock-determining parameters in a single block model and their recalculation in rock types (method of the recalculation depends on data type, classification type, etc.) seem to be a universal approach to automatic geological mapping. The approach is rather simple and its results seem to be geologically correct and plausible.

## Materials and methods

28 drill holes with an average depth of 580 m were sampled (Fig. 2). The sampling began at 420–500 m. A sample of the sedimentary cover was not taken. In total, there were 1029 samples of the drill cores with an average length of 4 m. Fe_tot_, Fe_2_O_3_, FeO, SiO_2_, Al_2_O_3_, P_2_O_5_, CaO, MgO, MnO, S_tot_, TiO_2_ were analyzed in the samples by spectrophotometric, atomic absorption spectrometric, and titration methods using the Agilent 8567, DL-22, SF-26, S-302 equipment at JSC “Belgorodgeologiya” (Belgorod, Russia) and “Voronezhgeologiya Ltd.” (Voronezh, Russia). Statistical investigations were carried out by the STATISTICA 12 program (StatSoft, https://www.statsoft.ru). Geostatistical studies, interpolation, and 3D modelling were conducted by the MINEFRAME 8 program (Mining Institute of Kola Science Centre, Russian Academy of Sciences, https://www.mineframe.ru) and Micromine 2016.1 (Micromine Pty Ltd., Australia, https://www.micromine.com; commercial license). Vector graphics were drawn by the Adobe Illustrator CS6, https://ww.adobe.com.

## Supplementary information

Supplementary Information.
