# Supplementary material for: Unsupervised geochemical classification and automatic 3D mapping of the Bolshetroitskoe high-grade iron ore deposit (Belgorod Region, Russia)
Source: Sci Rep. 2020 Oct 20;10:17861. doi: 10.1038/s41598-020-74505-y (PMC7576791; doi:10.1038/s41598-020-74505-y)
Supplement: Supplementary file 1 — Supplementary Information. [file 41598_2020_74505_MOESM1_ESM.docx]

‘С’ code of the artificial neural network used for the classification of rocks of the Bolshetroitskoe deposit

Andrey O. Kalashnikov^1,*^, Ivan I. Nikulin^2^, Dmitry G. Stepenshchikov^1^

^1^ Geological Institute of Kola Science Centre of Russian Academy of Sciences (GI KSC RAS), 14 Fersman Street, Apatity, Murmansk Region, 184209 Russia.

^2^ Norilskgeologia Ltd., 11 Grazhdanskiy Pr., Saint Petersburg, 195220 Russia

*Corresponding author. Email: [kalashnikov@geoksc.apatity.ru](mailto:kalashnikov@geoksc.apatity.ru)

A supplementary material of the article:

Kalashnikov A.O., Nikulin I.I., Stepenshchikov D.G. 2020. Unsupervised geochemical classification and automatic 3D mapping of the Bolshetroitskoe high-grade iron ore deposit (Belgorod Region, Russia). Scientific Reports, doi: 10.1038/s41598-020-74505-y

This file contains code of the artificial neural network used for the classification of rocks of the Bolshetroitskoe deposit. The code was generated by STATISTICA 12 (StatSoft, [www.statsoft.ru](http://www.statsoft.ru)) in .c format.

5 rock-forming components were used: Fe_2_O_3_, FeO, SiO_2_, Al_2_O_3_, CaO. Type of artificial neural network: Kohonnen’s network. Number of clusters: 12. Learning parameters: random sample sizes, train 70%, test 15%, validation 15%, seed for sampling is 1000, missing deleting handling (inputs) is casewise. Topological height 2, topological width 6. Comparison measure: Euclidian distance. 1000 training cycles. Learning rates: start 0.1, end 0.02. Neighborhoods: start 3, end 0. Normal randomization of network.

#include <stdio.h>

#include <conio.h>

#include <math.h>

#include <stdlib.h>

double ANN_Bolshetroitskoe_input_hidden_weights[12][5] =

{

{1.01686515528055e-001, 3.52161678796140e-001, 3.56422319541516e-001, 6.39948544504107e-001, 5.24042720499721e-002 },

{4.87026457227775e-001, 1.73951503029022e-001, 6.68330434129241e-001, 1.90275096732224e-002, 4.61475186836563e-002 },

{5.63846604732730e-001, 7.23859370373631e-001, 4.83394817765305e-002, 4.92446042679991e-002, 1.50124356224667e-001 },

{6.75763881174271e-001, 2.29376036143053e-001, 6.46538962993847e-002, 1.76738456712201e-002, 4.46499430263108e-001 },

{8.43577817149581e-001, 3.31717938423414e-001, 4.00043529277012e-002, 2.03948558649147e-002, 2.75299093309459e-002 },

{9.41432580305325e-001, 6.99845016720641e-002, 3.19919138710804e-002, 1.06944055900619e-002, 4.15321454514272e-002 },

{1.06306493963461e-001, 9.54546439578421e-002, 7.78408273201844e-001, 3.79266756228732e-001, 2.84663646505506e-002 },

{1.92556650007214e-001, 7.27548857300852e-001, 2.99179774216656e-001, 3.89355498758229e-001, 8.80823420383184e-002 },

{4.47563849071464e-001, 2.70971285944595e-001, 1.13393440459212e-001, 3.90069873974340e-001, 1.83713288943682e-001 },

{7.11185636604446e-001, 4.90378002376470e-001, 6.77164292029803e-002, 5.29837021009588e-002, 6.50956983042809e-002 },

{8.98150013386773e-001, 1.69721974469074e-001, 3.67759483149990e-002, 1.49733073404984e-002, 5.01599928340410e-002 },

{7.80022395374080e-001, 1.28206535100174e-001, 2.23822322542810e-001, 2.10230833335131e-002, 5.81368727441401e-002 }

};

double ANN_Bolshetroitskoe_max_input[5]={ 9.66424988100000e+001, 2.89500000000000e+001, 6.11700000000000e+001, 5.97000000000000e+001, 2.26700000000000e+001 };

double ANN_Bolshetroitskoe_min_input[5]={ 1.49771336000000e+000, 5.50000000000000e-001, 4.20000000000000e-001, 8.00000000000000e-002, 4.00000000000000e-002 };

double ANN_Bolshetroitskoe_input[5];

double ANN_Bolshetroitskoe_output[12];

long ANN_Bolshetroitskoe_winner;

int ANN_Bolshetroitskoe_position[2];

double ANN_Bolshetroitskoe_MeanInputs[5]={ 7.20433906826675e+001, 7.36900305131761e+000, 9.39741054091539e+000, 4.07251040221914e+000, 1.65632454923717e+000 };

void ANN_Bolshetroitskoe_ScaleInputs(double* input, double minimum, double maximum, int size)

{

double delta;

long i;

for(i=0; i<size; i++)

{

delta = (maximum-minimum)/(ANN_Bolshetroitskoe_max_input[i]-ANN_Bolshetroitskoe_min_input[i]);

input[i] = minimum - delta*ANN_Bolshetroitskoe_min_input[i]+ delta*input[i];

}

}

void ANN_Bolshetroitskoe_ForwardPropagate(double* invec,double* activations)

{

long i, j, k=1;

double dmin=0.0;

for(i=0; i<12; i++)

{

activations[i] = 0.0;

for(j=0; j<5; j++)

{

activations[i] += (ANN_Bolshetroitskoe_input_hidden_weights[i][j] - invec[j])*(ANN_Bolshetroitskoe_input_hidden_weights[i][j] - invec[j]);

}

activations[i] = sqrt(activations[i]);

if(activations[i]<dmin || i==0)

{

dmin = activations[i];

ANN_Bolshetroitskoe_winner = i;

}

}

for(i=1; i<=2; i++)

{

ANN_Bolshetroitskoe_position[0] = i;

for(j=1; j<=6; j++)

{

ANN_Bolshetroitskoe_position[1] = j;

if(k<ANN_Bolshetroitskoe_winner+1) k++;

else goto EXIT;

}

}

EXIT:;

}

int main()

{

int cont_inps;

int i=0;

int keyin=1;

while(1)

{

printf("\n%s\n","Enter values for Continuous inputs (To skip a continuous input please enter -9999)");

printf("%s","Cont. Input-1(Fe2O3*коэф): ");

scanf("%lg",&ANN_Bolshetroitskoe_input[0]);

printf("%s","Cont. Input-2(FeO): ");

scanf("%lg",&ANN_Bolshetroitskoe_input[1]);

printf("%s","Cont. Input-3(SiO2): ");

scanf("%lg",&ANN_Bolshetroitskoe_input[2]);

printf("%s","Cont. Input-4(Al2O3): ");

scanf("%lg",&ANN_Bolshetroitskoe_input[3]);

printf("%s","Cont. Input-5(CaO): ");

scanf("%lg",&ANN_Bolshetroitskoe_input[4]);

for(cont_inps=0;cont_inps<5;cont_inps++)

{

//Substitution of missing continuous variables

if(ANN_Bolshetroitskoe_input[cont_inps] == -9999)

ANN_Bolshetroitskoe_input[cont_inps]=ANN_Bolshetroitskoe_MeanInputs[cont_inps];

}

ANN_Bolshetroitskoe_ScaleInputs(ANN_Bolshetroitskoe_input,0,1,5);

ANN_Bolshetroitskoe_ForwardPropagate(ANN_Bolshetroitskoe_input,ANN_Bolshetroitskoe_output);

printf("\nWinner = (%d,%d)",ANN_Bolshetroitskoe_position[0],ANN_Bolshetroitskoe_position[1]);

printf("\nActivation = %.14e",ANN_Bolshetroitskoe_output[ANN_Bolshetroitskoe_winner]);

printf("\n\n%s\n","Press any key to make another prediction or enter 0 to quit the program.");

keyin=getch();

if(keyin==48)break;

}

return 0;

}
